# Adenocarcinoma of the jejunum: management of a rare small bowel neoplasm

**DOI:** 10.1093/jscr/rjab124

**Published:** 2021-04-22

**Authors:** Raquel Pereira, André Tojal, Aline Gomes, Carlos Casimiro, Sara Moreira, Fortunato Vieira, Pedro Rodrigues

**Affiliations:** 1 General Surgery Department, Tondela-Viseu Hospital Centre, Viseu, Portugal; 2 Oncology Department, Tondela-Viseu Hospital Centre, Viseu, Portugal; 3 Pathology Department, Histocit Laboratory, Trofa, Portugal; 4 Pathology Department, Tondela-Viseu Hospital Centre, Viseu, Portugal

## Abstract

Small bowel adenocarcinomas are rare malignant tumors that account for less than 2% of gastrointestinal tumors. Despite a thorough history, physical examination and complete diagnostic workup, the correct diagnosis of small intestinal neoplasm has been established preoperatively in only 50% of cases. Due to the rarity of this disease, there are very few established guidelines for its management and it has been primarily treated the same way as colorectal cancer, even though patient’s prognostic outcome is worse. With new guidelines in 2020, we review a clinical case of a 64-year-old male patient with adenocarcinoma of the jejunum treated in our institution.

## INTRODUCTION

Small bowel adenocarcinomas (SBA) are rare malignant tumors that account for less than 2% of gastrointestinal tumors [1]. Adenocarcinoma comprises an estimated 30–40% of small bowel neoplasms and is most commonly located in the duodenum (57%), while 29% of cases are located in the jejunum and 10% in the ileum [2, 3]. According to EUROCARE, the incidence of SBA is estimated at 3595 new cases per year in Europe [4]. Due to the rarity of this disease, there are very few established guidelines for its management and it has been primarily treated the same way as colorectal cancer, even though patient’s prognostic outcome is worse.

With new guidelines in 2020, we review a clinical case of adenocarcinoma of the jejunum [5].

This work was reported in line with the SCARE criteria [6].

## CASE REPORT

The patient was a 64-year-old Caucasian male presenting at our emergency room (ER) with progressive asthenia, adynamia and paleness for about 1 month. Gastrointestinal bleeding was not reported.

Past relevant medical history included colonic polypectomy 2 years prior. The patient had no symptoms at that time, total colonoscopy was performed as part of colorectal cancer screening program and revealed two polyps—tubular adenomas with low grade dysplasia. Relevant family history included one brother with gastric neoplasia.

Laboratory tests revealed severe microcytic anemia (Hemoglobin 4.8 g/dl).

After stabilization, the patient underwent upper gastrointestinal endoscopy and colonoscopy, both with no alterations.

Therefore, a capsule endoscopy was obtained which revealed an ‘obstructing, large, friable and hemorrhagic tumor of proximal jejunum’ and subsequent single-balloon enteroscopy was performed but it was not possible to reach the neoplasia.

**Figure 1:**
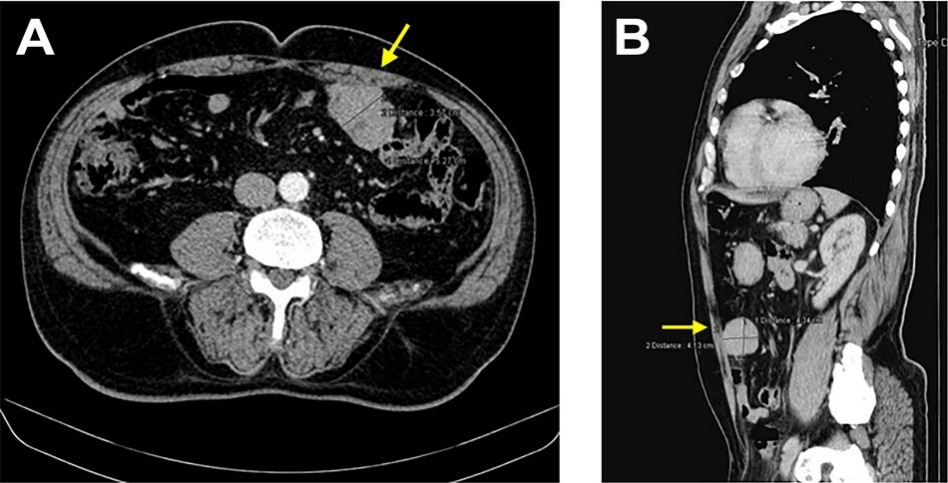
CT images showing a nodular lesion with 48 × 57 × 44 mm, close to the anterior abdominal wall (yellow arrow).

The staging computed tomography (CT) revealed a ‘nodular image with 48 × 57 × 44 mm, close to the anterior abdominal wall’ (Fig. 1) with active bleeding, two non-specific nodules in the left lung and five in the right lung, all infracentimetric.

The patient underwent urgent laparoscopic segmental intestinal resection and omental lymph node resection. The lesion was a 5-cm mass at proximal jejunum without serosal invasion. Enterectomy was performed with 5 cm proximal and distal margins and end-to-end small intestinal manual anastomosis.

No complications were reported and the patient was discharged one week after surgery.

**Figure 2:**
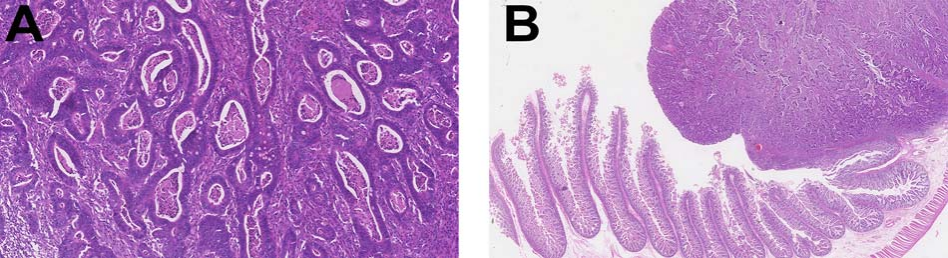
Histopathology examination revealed invasive adenocarcinoma moderately differentiated of small bowel. The lesion invaded almost all *tunica muscularis* with little stromal desmoplastic reaction and moderate polymorph inflammatory response (left—H&E 20×; right—H&E 1.25×). There was no evidence of lymphatic or venous invasion. Of the five lymph nodes examined, no metastasis were found. Free surgical margins were achieved.

Histopathology examination confirmed invasive adenocarcinoma moderately differentiated with no evidence of metastasis on five lymph nodes examined—pT2N0M0, stage I disease—and free surgical margins (Fig. 2).

Due to the lack of clinical guidelines at that time (2015), the team decided on clinical management similar to colon adenocarcinoma.

No genetic testing was performed since there were no clear guidelines.

The patient was followed up for a period of 5 years during which his medical history was collected with physical examination, CEA and CA 19.9 were evaluated every 6 months and chest, abdominal and pelvic CT scans performed every year. A total Colonoscopy with Ileoscopy was performed 3 years after surgery and a 6 mm sigmoid polyp was removed—tubular adenoma with low grade dysplasia.

**Figure 3:**
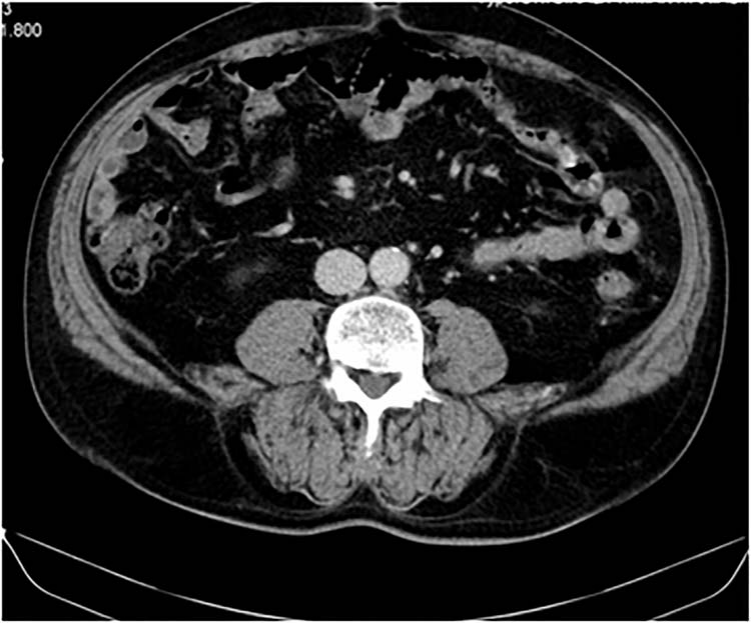
CT image at 1-year follow up showing no local tumor recurrence.

No complications or remission signs were found, lung nodules remained stable and the patient remained asymptomatic (Fig. 3).

## DISCUSSION

SBA is extremely rare. Despite a complete diagnostic workup, the correct diagnosis has been established preoperatively in only 50% of cases [7].

The diagnosis is delayed because symptoms are non-specific and small intestine is not totally accessible to endoscopic examination. It usually manifests through complications like occlusion (40%) or bleeding (24%) [2].

Since SBA is often diagnosed at advanced stages, the outcome is generally poor with a 5-year survival of less than 30% and median survival of 19 months [1].

Historically, management of SBA has been extrapolated on the treatment approach for large bowel adenocarcinoma [8]. Risk factors for SBA are similar to those for colorectal cancer: lifestyle factors and predisposing diseases like familial adenomatous polyposis, hereditary non polyposis colorectal cancer (Lynch syndrome), Peutz-Jeghers syndrome, Crohn’s disease and celiac disease [1]. However, recent molecular studies show genomic differences between these tumors and suggest new future pathways for its treatment [8].

The understanding of this rare cancer warranted the establishment of specific guidelines, like French intergroup clinical practice guidelines (2018) and NCCN Clinical Practice Guidelines in Oncology (2019). Both agree in surgical resection as the curative treatment, depending on factors related to the tumor and the patient. For adenocarcinoma located in the jejunum, segmental resection with lymph node dissection is recommended [1, 5]. In stage I, like in our clinical case, treatment recommendation is surgery only.

No studies have evaluated adjuvant therapy after resection. A prospective international Phase III study comparing adjuvant chemotherapy vs observation is currently underway (PRODIGE 33-BALLAD study; NCT02502370) [1, 5].

NCCN guidelines recommend MMR or MSI testing for all patients with SBA because it can function as a prognostic and/or predictive marker and can help identify patients who should be tested for Lynch syndrome [5].

NCCN recommendations for adjuvant therapy are applied to stage II and III. In stage II, the adjuvant therapy is dependent on MSI-H or dMMR and high-risk features (T4 stage, close or positive surgical margins, few lymph nodes examined—<5 for duodenal or <8 for jejunal/ileal primary tumor location—or tumor perforation).

Patients with locally unresectable disease may undergo neoadjuvant therapy and should be routinely monitored for conversion to resectable disease.

The main sites of distant metastasis are liver and peritoneal cavity. It should be considered metastasectomy in resectable metastasis, surgical citoreduction with hyperthermic intraperitoneal chemotherapy and systemic therapy [1, 5].

Follow-up is done during the first 5 years and includes clinical history, physical examination, CEA and/or CA 19–9 measurement and chest, abdominal and pelvic CT scans.

## CONCLUSION

The diagnosis of SBA is challenging and frequently delayed.

Symptoms are non-specific and it cannot be totally accessed without the use of capsule endoscopy or double-balloon enteroscopy.

According to National Comprehensive Cancer Network (NCCN) Guidelines version 1.2020, for local (stages I–III) SBA, like in our clinical case, primary treatment consists of surgical resection with en bloc removal of the regional lymph nodes. These recent guidelines also recommend MMR or MSI testing for all patients with SBA.

Recent specifics guidelines provide us uniformization of clinical care.

## CONFLICT OF INTEREST STATEMENT

None declared.
